# Impact of neoadjuvant chemotherapy on somatic mutation status in high-grade serous ovarian carcinoma

**DOI:** 10.1186/s13048-022-00983-5

**Published:** 2022-05-02

**Authors:** Zibi Marchocki, Alicia Tone, Carl Virtanen, Richard de Borja, Blaise Clarke, Theodore Brown, Taymaa May

**Affiliations:** 1 Department of Surgical Oncology, Division of Gynecologic Oncology, Princess Margaret Cancer Centre, University Health Network, Toronto, ON Canada; 2 Department of Obstetrics and Gynecology, University of Toronto, Toronto, ON Canada; 3 Bioinformatics and HPC Services Core, Princess Margaret Cancer Centre, Toronto, Ontario Canada; 4 Department of Pathology, University Health Network, Toronto, Ontario Canada; 5 The Lunenfeld-Tanenbaum Research Institute, Mount Sinai Hospital, Toronto, Ontario Canada

**Keywords:** Neoadjuvant chemotherapy, High-grade serous ovarian cancer, Platinum resistance, Non-synonymous gene mutation

## Abstract

**Background:**

Patients treated with neoadjuvant chemotherapy (NACT) for advanced high-grade serous ovarian carcinoma (HGSC) have a higher rate and shorter time to platinum-resistant recurrence compared to patients treated with primary cytoreductive surgery (PCS) and adjuvant chemotherapy. The purpose of this study is to determine the impact of NACT on somatic mutation status in platinum-sensitive and resistant HGSC. Patients with advanced HGSC who had a documented response to platinum-based NACT, a banked blood sample, and a banked tumor sample before and after NACT were identified. Whole exome and/or targeted deep sequencing was performed in matched normal and pre/post-NACT tumor samples from 3 platinum-resistant and 2 platinum-sensitive patients to identify somatic non-synonymous mutations at each time point.

**Results:**

When comparing exonic non-synonymous mutations in pre-NACT and post-NACT samples from the same patient, an average of 41% (1-68%) of genes were mutated at both time points. There were no trends detected in the mutational burden following exposure to NACT in platinum-resistant vs. platinum-sensitive cases. The majority of mutated genes were unique to each case. We identified several genes that were commonly mutated in pre-NACT samples specific to platinum-resistant (*CSPG4, SLC35G5, TUBA3D*) or sensitive (*CYP2D6, NUTM1*, *DNAH5*) cases. Four mutated genes emerged exclusively in the platinum-resistant cases (*ADGRV1, MUC17, MUC20, PAK2*) following NACT.

**Conclusions:**

Patients with advanced HGSC present with significant intra-tumor heterogeneity. NACT significantly impacts the somatic mutation status irrespective of the time to recurrence. The mutated genes detected in chemo-naive pre-NACT tumor samples from either resistant or sensitive cases could potentially have a role in the prediction of chemotherapy response in patients scheduled to receive NACT; larger studies are required to further validate these genes.

**Supplementary Information:**

The online version contains supplementary material available at 10.1186/s13048-022-00983-5.

## Background

Epithelial ovarian cancer is the most lethal cancer of the female reproductive system in the developed world [1]. As 70% of patients present with advanced disease, the prognosis remains poor with high mortality and a 5-year survival of 48.6% [1]. The most common histological subtype is high-grade serous ovarian cancer (HGSC), which accounts for 90% of stage III/IV cases and 70% of ovarian cancer cases overall [2]. The standard treatment for HGSC includes primary cytoreductive surgery (PCS) and post-operative platinum- and taxane-based chemotherapy [3]. Residual disease after PCS is a major prognostic factor, with the highest survival noted in those patients who undergo a complete cytoreduction to no visible disease [4]. Patients with multiple comorbidities, poor functional status, or where the likelihood of optimal cytoreduction is low, may be considered for neoadjuvant chemotherapy (NACT) followed by interval cytoreductive surgery (ICS) as an alternative option to PCS [3]. Treatment with NACT and ICS has several advantages over PCS including less extensive surgery and reduced perioperative morbidity [5]. The rate of complete cytoreduction is higher in patients undergoing ICS compared to PCS; however, this has not been shown to provide any survival benefit [5]. This has raised concerns regarding the negative impact of NACT and its effect on platinum-resistant recurrence [6]. In addition to increasing the rate of platinum-resistant recurrence, NACT also shortens the time to platinum-resistant recurrence [6]. The response rate to subsequent chemotherapy for all patients with HGSC who develop-platinum resistance is low (15%) with a progression-free survival of 3-4 months and a median survival of less than a year [7].

Although development of platinum resistance following NACT is poorly understood, several possible mechanisms have been suggested. The introduction of NACT to a large volume of tumor pre-operatively may increase the enrichment of the platinum-resistant subclones that are already present at the time of diagnosis [8]. In contrast, this large tumor burden at presentation would be removed in patients undergoing PCS prior to treatment with adjuvant systemic chemotherapy. The limited blood supply of the large tumors may upregulate Hypoxia-inducible factor 1-alpha (HIF-1α), a hypoxia-related transcription factor linked to platinum-resistance [9]. Furthermore, operative surgical planes undergo a treatment effect, making an assessment of optimal excision at ICS challenging [10]. Inadequate tumor resection at ICS combined with NACT-induced stemness of ovarian cancer cells in the residual tumor [11] may result in a faster emergence of platinum resistance, and as a result a decreased survival in comparison to patients treated with PCS. Lastly, NACT has been shown to induce gene mutations such as *TP53* and K351N, which are associated with shorter disease-free survival [12].

In this study, we examined the effects of NACT on the somatic mutation status in matched tumor samples obtained pre and post-NACT in women with HGSC to determine if NACT alters/enriches non-synonymous mutations within tumors and to identify candidate mutations that associate with platinum resistance.

## Results

A total of 5 cases of stage IIIa-IIIc HGSC treated with 3-5 cycles of platinum-based NACT were selected (Table 1). This included three cases with platinum-resistant (Case 1-3) and two with platinum-sensitive disease (Case 4-5). The biochemical response in terms of reduction of Ca125 level ranged from 66 to 84% for platinum-resistant cases and 96 to 98% for platinum-sensitive cases. The average time to recurrence was 4.2 months in platinum-resistant, and 20.2 months in platinum-sensitive cases. All women with platinum-resistant HGSC died of their disease within 12.1-21.4 months from diagnosis, while both patients with platinum-sensitive HGSC were alive at the last follow-up 42.2 and 47.9 months after the diagnosis.

**Table 1:** Summary of study cases and samples

#	Stage	NACT cycles^a^	CA-125 Response^b^	Residual Disease at Interval Surgery	Time to Recurrence^c^	Time to Death^d^	Tumor Samples *(ID)*
1	IIIa	5 (9)	81 (84%)	0 cm	4.6 (Resistant)	21.4 (DOD)	**Pre:** omental biopsy ***(1-1)***^a,b^ **Post:** pelvic nodule ***(1-2)***^a^, omentum ***(1-3)***^b^
2	IIIc	5 (5)	180 (62%)	> 1 cm	4.4 (Resistant)	12.1 (DOD)	**Pre:** omental biopsy ***(2-1)***^a,b^ **Post:** omentum ***(2-2)***^a,b^
3	IIIb	4 (7)	96 (82%)	0 cm	3.6 (Resistant)	18.1 (DOD)	**Pre:** omental biopsy ***(3-1)***^a,b^ **Post:** right ovary ***(3-2)***^a,b^, omentum ***(3-3)***^a,b^
4	IIIb	3 (6)	1634 (98%)	> 1 cm	19.5 (Sensitive)	*47.9 (AWD)*	**Pre:** omental biopsy ***(4-1)***^a,b^ **Post:** stomach nodule ***(4-2)***^a,b^
5	IIIc	3 (6)	4659 (96%)	< 1 cm	20.9 (Sensitive)	*42.2 (AWD)*	**Pre:** biopsy (not specified) ***(5-1)***^a^ **Post:** bowel nodule ***(5-2)***^a^

All patients had stage IIIa-c high-grade serous ovarian carcinoma that was treated with neo-adjuvant intravenous Carboplatin/Taxol chemotherapy (NACT) prior to interval cytoreductive surgery. Tumor samples were obtained at diagnosis (prior to NACT, “Pre”) and at surgery (after 3-5 cycles of NACT, “Post”). Buffy coat samples served as normal controls

^a^total first-line cycles indicated in brackets

^b^CA-125 levels prior to the first cycle of NACT, with the percentage decrease after NACT indicated in brackets

^c^time to recurrence is shown in months and is based on the first observation of radiologic progression, typically with a concomitant or preceding rise in serum CA-125

^d^time to death or last follow-up is shown in months, with final status in brackets (“DOD” = dead of disease, “AWD” = alive with disease). Neither of the platinum-sensitive cases had developed platinum-resistant disease at last follow-up

Tumor sample sites included in whole exome sequencing (“a”) and targeted deep sequencing (“b”) are indicated

Whole exome sequencing was performed in buffy coat and macro-dissected pre-NACT (*N* = 5 samples) and post-NACT tumor (*N* = 6 samples). A summary of the somatic mutation counts is shown in Table 2 (exonic) and Additional File 1 (non exonic). When comparing exonic non-synonymous mutations in pre-NACT and post-NACT samples from the same patient, an average of 41% (1-68%) of genes were mutated at both time points (Table 2). There were no visible trends in increase vs. decrease in the mutational burden following exposure to NACT in resistant vs. sensitive cases.

**Table 2:** Summary of somatic mutation counts by whole-exome sequencing

Case	Sample ID	Total Somatic Mutations	Exonic Mutations	Synonymous SNV	Non- synonymous SNV	stoploss/ stopgain	Shared pre + post-NACT (position)^a^	Shared pre + post-NACT (gene)^b^
1 (R)	**1-1** (pre)	333	71	24	**47**	**0**	**13%** (6/47)	**17%** (8/47)
	**1-2** (post)	5652	1735	904	**821**	**10**	**1%** (6/831)	**1%** (8/691)
2 (R)	**2-1** (pre)	427	116	34	**77**	**5**	**41%** (34/82)	**41%** (33/80)
	**2-2** (post)	543	120	45	**73**	**2**	**45%** (34/75)	**51%** (33/65)
3 (R)	**3-1** (pre)	399	65	25	**38**	**2**	**65%**^**c**^ (26/40)	**68%**^**c**^ (27/40)
	**3-2** (post)	351	61	27	**32**	**2**	**62%**^**d**^ (21/34)	**64%**^**d**^ (21/33)
	**3-3** (post)	298	55	23	**31**	**1**	**41%**^**d**^ (13/32)	**47%**^**d**^ (14/30)
4 (S)	**4-1** (pre)	539	128	33	**88**	**7**	**43%** (41/95)	**44%** (42/95)
	**4-2** (post)	510	137	39	**92**	**6**	**42%** (41/98)	**43%** (42/98)
5 (S)	**5-1** (pre)	1412	436	216	**211**	**9**	**16%** (35/220)	**17%** (35/209)
	**5-2** (post)	703	106	35	**69**	**2**	**49%** (35/71)	**58%** (35/60)

Total number of somatic mutations detected in pre and post-NACT tumor samples using whole-exome sequencing. The numbers of exonic and non-synonymous exonic mutations (including mutations classified as non-synonymous SNV, stopgain or stoploss) are highlighted, in addition to the overlap of non-synonymous mutations in pre-NACT and post-NACT samples from the same patient

^a^comparison made based on genomic position

^b^comparison made based on gene name

^**c**^mutations shared with at least one post-NACT sample from same case

^**d**^mutations shared with pre-NACT sample from same case

*Abbreviations: NACT*  Neoadjuvant chemotherapy; *R *platinum-resistant case, S - platinum-sensitive case, *SNV *Single nucleotide variant

**Fig. 1:**
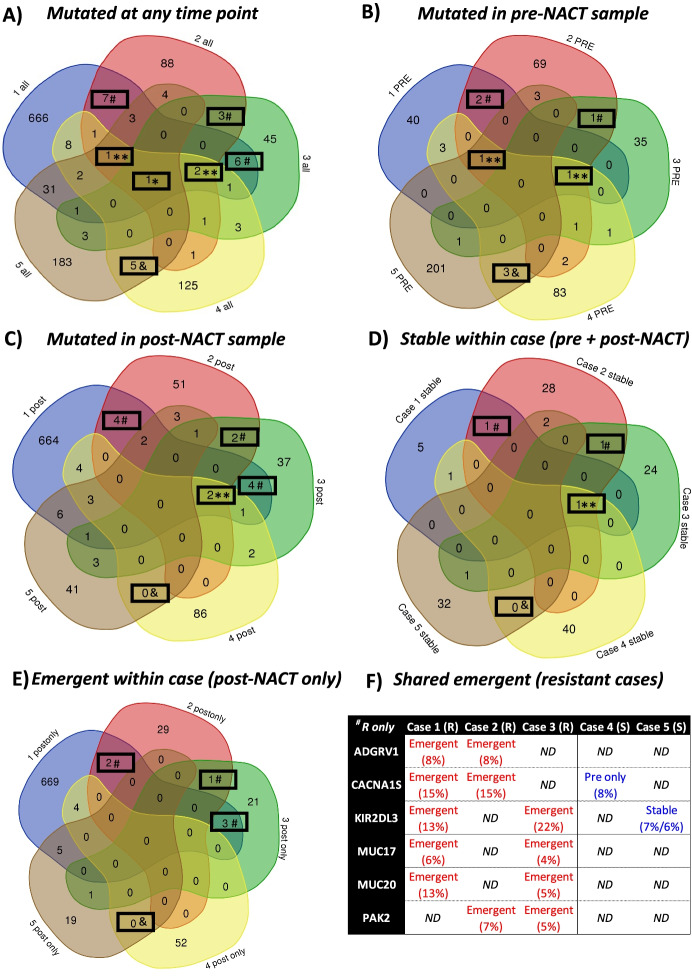
Comparison of mutated genes among all sequenced cases. Comparisons of genes with non-synonymous exonic mutations among all cases are shown. Genes in panel **(A)** include those mutated in samples obtained pre-NACT, post-NACT or at both time points, with only pre-NACT or post-NACT samples included in panels **(B)** and **(C)** respectively. Panel **(D)** includes genes mutated in pre-NACT and post-NACT samples from the same patient (stable within case), while panel **(E)** includes genes mutated in only post-NACT samples from the same patient (emergent within case). Emergent genes in > 1 resistant cases are highlighted in panel **(F)**. The allele fraction of the detected mutation is shown (reads for variant allele/total reads). * gene mutated in 5/5 cases; **gene mutated in 4/5 cases; ^#^gene mutated in > 1 resistant cases but not in sensitive cases; ^&^gene mutated in both sensitive cases. *Abbreviations: ND* No mutation detected, *R* Resistant case, *S* Sensitive case

The comparison of genes with non-synonymous exonic mutations detected at any time point (pre-NACT, post-NACT, or both) is shown in Fig. 1A. The majority of mutated genes were unique to each case, including 666/730 (91%) in Case 1, 88/112 (79%) in Case 2, 45/66 (68%) in Case 3, 125/151 (83%) in Case 4 and 183/234 (78%) in Case 5. All recurrently mutated genes (> 1 case) are listed in Additional File 2. Of note, all cases had detected mutations in *MUC2*, while mutations in *DDX11*, *TP53* and *TUBA3D* were observed in 4 of 5 cases. Genes that were exclusively mutated in > 1 resistant or sensitive cases are highlighted in Table 3; 16 genes were exclusively mutated in > 1 resistant case and 5 genes were exclusively mutated in both sensitive cases.

**Table 3:** Summary of recurrently mutated genes in only resistant or sensitive cases by whole-exome sequencing

Gene Symbol	Case 1 (R)		Case 2 (R)		Case 3 (R)		Case 4 (S)		Case 5 (S)	
	Pre	Post	Pre	Post	Pre	Post	Pre	Post	Pre	Post
*Genes mutated in > 1 platinum-resistant cases only (16)*										
*ADGRV1/GPR98^a^		**X**		**X**						
*AOC1/ABP1^a^		**X**			**X**	**X**^**e**^				
ARHGAP5			**X**			**X**^**e**^				
*CSPG4^a^	**X**		**X**	**X**						
KIR2DL1		**X**			**X**					
KRTAP4-11	**X**					**X**^**e**^				
MMP9		**X**	**X**							
*MTMR11^c^		**X**			**X**	**X**^**d**^				
MUC17^c^		**X**				**X**^**e**^				
MUC20^c^		**X**				**X**^**e**^				
*OR52N5^c^		**X**	**X**	**X**						
PAK2^c^				**X**		**X**^**e**^				
*PCDHB11^c^			**X**	**X**		**X**^**e**^				
*TMEM14B^c^		**X**	**X**	**X**						
TTN		**X**	**X**							
USP8		**X**	**X**							
*Genes mutated in > 1 platinum-sensitive cases only (5)*										
*CYP2D6^b^							**X**		**X**	
*DNAH5^b^							**X**	**X**	**X**	
FAM186A								**X**	**X**	
MACF1								**X**	**X**	
NUTM1^b^							**X**		**X**	

Genes that were recurrently mutated in either resistant or sensitive cases (irrespective of time point) are shown. Whether a non-synonymous exonic mutation was detected in pre-NACT and/or post-NACT samples from each case is indicated by an “X”. Specific mutations are included in Additional File 3. *included on targeted panel; ^**a**^gene mutated in pre-NACT samples from > 1 resistant cases but not sensitive cases (*CSPG4*); ^**b**^gene mutated in pre-NACT samples from both sensitive cases but not resistant cases (*CYP2D6, NUTM1, DNAH5*); ^**c**^gene mutated in post-NACT samples from > 1 resistant cases but not sensitive cases (*ADGRV1*, *AOC1*, *MTMR11*, *MUC17*, *MUC20*, *OR52N5*, *PAK2*, *PCDHB11*, *TMEM14B*); ^**d**^gene mutated in both omental and ovarian post-NACT samples from Case 3 *(MTMR11);* ^e^gene mutated in only 1 post-NACT sample from Case 3 *(AOC1, ARHGAP5, KRTAP4-11, MUC17, MUC20, PAK2, PCDHB11)*

The comparison of genes with non-synonymous exonic mutations detected within a given time point is shown in Fig. 1B (pre-NACT) and 1C (post-NACT). The majority of mutations were case-specific, including 85-96% among pre-NACT samples and 68-96% among post-NACT samples. However, a few genes were commonly mutated in pre-NACT samples in exclusively resistant (*CSPG4, SLC35G5, TUBA3D*) or sensitive (*CYP2D6, NUTM1, DNAH5*) cases. Among post-NACT samples, 10 genes were exclusively mutated in > 1 resistant case (*ADGRV1, AOC1, CACNA1S, MTMR11, MUC17, MUC20, OR52N5, PAK2, PCDHB11, TMEM14B*); no genes were exclusively mutated in both sensitive cases at this time point. As shown in Table 2 a small proportion of the same mutations were present at both time points (pre- and post-NACT) within the same case; the comparison of these stably mutated genes is shown in Fig. 1D. Of note, only 2 genes were shared between the resistant cases (*SLC35G5, TUBA3D*), while there were no shared genes between the sensitive cases. To compare the potential impact of NACT in our cases, we next looked at the genes that were mutated in post-NACT, but not pre-NACT samples, from the same case; this included 683/691 (99%) of post-treatment genes from Case 1, 32/65 (49%) from Case 2, 26/53 (49%) from Case 3, 56/98 (57%) from Case 4 and 25/60 (42%) from Case 5. The comparison of these emergent mutations is shown in Fig. 1E; of note, 6 genes were shared between the resistant cases (highlighted in Fig. 1F). While two of these genes showed a different pattern of mutation in sensitive cases (*CACNA1S* in pre-NACT only and *KIR2DL3* at a low level in both pre-NACT and post-NACT), the remaining 4 genes were exclusively mutated in platinum-resistant cases (*ADGRV1, MUC17, MUC20, PAK2*). There were no genes with treatment-emergent mutations shared between the two sensitive cases.

**Fig. 2:**
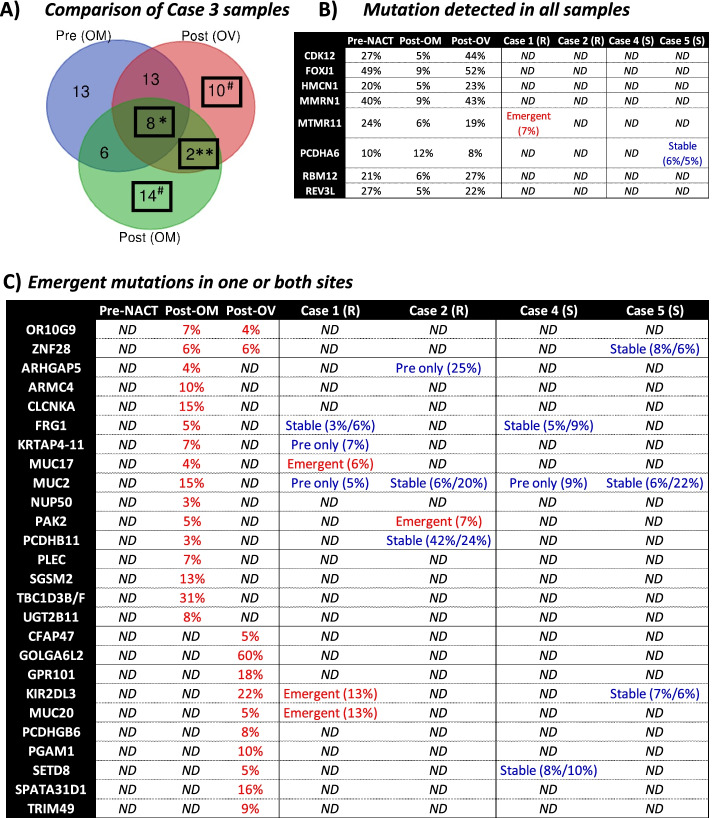
Comparison of mutated genes by tissue site and treatment status within a platinum-resistant Case 3. Panel **(A)** shows the comparison of genes mutated in the 3 samples obtained from Case 3, including pre-NACT and post-NACT samples from the omentum (OM) and a post-NACT sample from the ovary (OV). Genes with mutations in all samples are highlighted in panel **(B)**, while those with emergent mutations following NACT are highlighted in panel **(C)**. *mutated irrespective of tissue site and treatment site. **emergent in both post-NACT samples. ^#^emergent in one post-NACT sample. ND = not detected, R = resistant, S = sensitive

The inclusion of post-NACT samples from two different tumor sites (right ovary and omentum) for platinum-resistant Case 3 allowed us to compare the impact of both sites and exposure to NACT on mutational status within the same patient (Fig. 2A; see Additional File 3 for more detailed gene list). Of note, 8 genes were commonly mutated among all samples (highlighted in Fig. 2B; *CDK12, FOXJ1, HMCN1, MMRN1, MTMR11, PCDHA6, RBM12, REV3L*). The same mutation was detected in all samples, with no significant changes in allele fraction following NACT. Both omental samples had detected mutations in 6 genes irrespective of treatment status, while 2 genes had the same emergent mutations in both post-NACT samples irrespective of tissue site (*OR10G9, ZNF28*). There were also site-specific differences in post-treatment samples, including 10 genes with emergent mutations only at the ovarian site and 14 genes with emergent mutations only at the omental site (highlighted in Fig. 2C). While five of the genes with emergent mutations showed a different pattern of mutations in sensitive cases (*FRG1, KIR2DL3, MUC2, SETD8, ZNF28*), the majority were exclusive to platinum-resistant cases (*ARHGAP5, ARMC4, CFAP47, CLCNKA, GOLGA6L2, GPR101, KRTAP4-11, MUC17, MUC20, NUP50, OR10G9, PAK2, PCDHB11, PCDHGB6, PGAM1, PLEC, SGSM2, SPATA31D1, TBC1D3B/F, TRIM49, UGT2B11*).

**Fig. 3:**
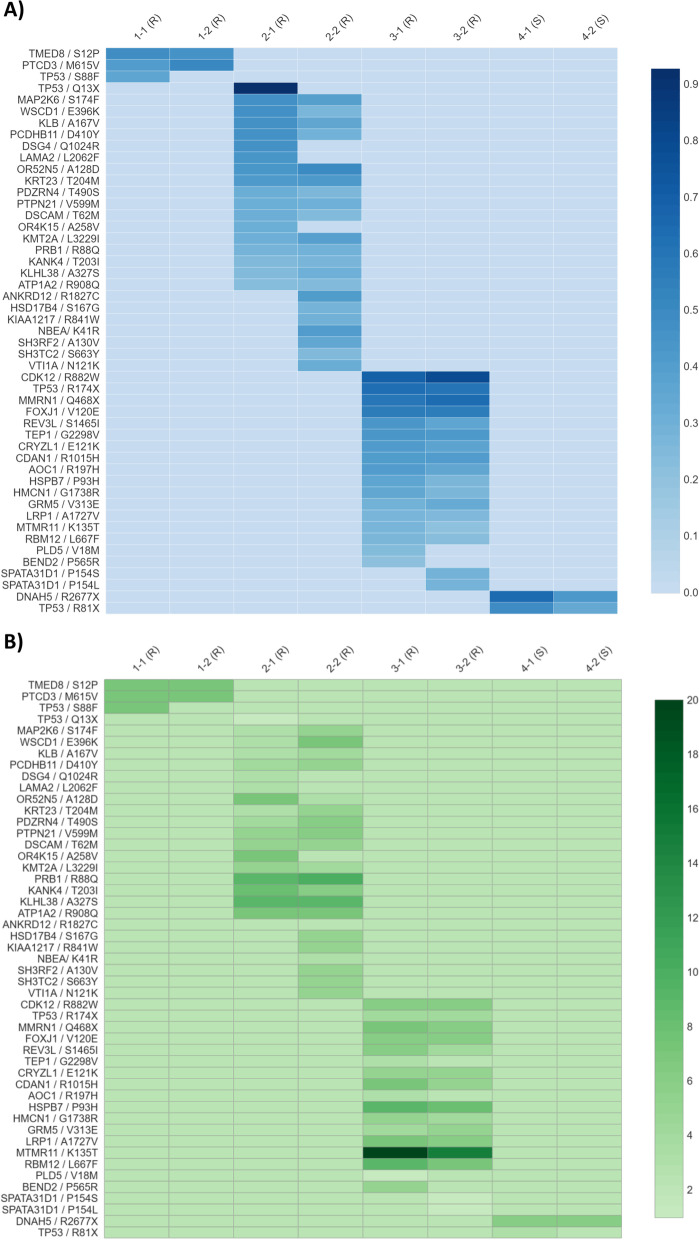
Select candidate mutations validated by targeted sequencing: allele fraction and copy number. Panel **(A)** shows the allelic fraction of mutations and panel **(B)** shows the tumor copy number for exonic non-synonymous mutations validated by our targeted sequencing panel. Each row represents a specific mutation (with gene name / amino acid change indicated at left) and each column represents a sample. Copy number at each site was 2 in all normal samples (not shown). R = resistant case; S = sensitive case

The results of targeted deep sequencing of 75 candidate genes selected from our exome data are summarized in Fig. 3 (see Additional File 4 for full panel gene list and selection criteria). Mutations were validated in several genes in platinum-resistant cases, while mutation of *DNAH5* was confirmed in both pre-NACT and post-NACT samples from Case 4, the one platinum-sensitive case with sufficient material for targeted sequencing (Fig. 3A). Of note, emergent mutations in post-treatment samples were confirmed in seven genes in platinum-resistant Case 2 (*ANKRD12, HSD17B4, KIAA1217, NBEA, SH3RF2, SH3TC2, VTI1A*) and one gene in platinum-resistant Case 3 (*SPATA31D1*). Copy number changes were also observed in several genes prior to and/or following NACT (Fig. 3B). Of note, copy numbers of ≥7 were observed in 2 genes in Case 1 (*TMED8, PTCD3*), six genes in Case 2 (*WSCD1, OR52N5, PRB1, KANK4, KLHL38, ATP1A2*), and six genes in Case 3 (*MMRN1, CDAN1, HSPB7, LRP1, MTMR11, RBM12*). Furthermore, copy number loss was observed in *SPATA31D1* following NACT in Case 4 only.

## Discussion

Studies have demonstrated that HGSC is a multi-clonal disease with a high degree of genomic instability and extensive intra-tumor heterogeneity in functional cellularity [13], copy number alterations [14], and somatic mutation status [15]. In line with these studies, the majority of the mutated genes in our study were unique to each case. In addition, the large heterogeneity of mutation profiles was also noted within the same case between different tumour sites. Similar findings were demonstrated by Bashashati and colleagues who used whole exome, targeted deep sequencing, and copy number analysis to reveal extensive diversity in 31 spatially separated tumor samples obtained prior to treatment from 6 patients with HGSC [15]. Distinct clones were observed within and between tumor masses from the same patient, with an average of 52% (range 10-91%) of somatic mutations present in every sample [15]. Copy number and mutation profiles evolved independently. In the same study, the clonal evolution analysis in one matched primary/recurrent tumor pair revealed a much higher degree of genetic similarity than expected. Anecdotally, this case had a longer overall survival (74 months), which could suggest that clonal stability (especially following chemotherapy) results in an improved outcome [15]. We identified only 2 mutated genes which were shared between the resistant cases (*SLC35G5, TUBA3D*) pre-and-post-NACT, while there were no shared mutated genes between the sensitive cases. A study by Schwarz et al. used a whole-genome copy number analysis in 135 spatially and temporally separated samples from 14 patients predominantly treated by NACT [14]. Those with a high degree of clonal expansion had worse OS compared to patients with a low degree of clonal expansion, suggesting a relationship between heterogeneity and outcome. Comparison of pre-and-post-NACT samples from 10 patients revealed small alterations in heterogeneity at the CNV level. The extent of temporal heterogeneity was not predictive of survival; however, pre- and post-NACT samples were not site-matched [14]. Extensive intra- and inter- tumor heterogeneity highlight the importance of tumor sampling from many locations before making any decision about the treatment if we are to rely on the genetic information.

We found a significant impact of NACT on the somatic mutation status in all five patients, irrespective of the time to recurrence, based on the low percentage of shared mutations between pre- and post-NACT samples in all cases. However, there were no visible trends in mutational burden following the exposure to NACT in resistant vs. sensitive cases. Similar findings were demonstrated by Cooke et al. at the CNV level [8]. The authors used array comparative genomic hybridization to analyze six paired pre- and post-neoadjuvant treatment HGSC samples from the CTCR-OV01 clinical study. The results did not show extensive copy number differences, suggesting that platinum resistance develops from pre-existing minor clones.

In this study, we have identified a few commonly mutated genes in pre-NACT tumor samples from either sensitive (*DNAH5, CYP2D6, NUTM1*) or resistant (*CSPG4, TUBA3D, SLC35G5*) cases, which suggest that these genes could have a potential utility for prediction of the response to NACT. However, this would have to be validated in a larger cohort of patients.

Dynein Axonemal Heavy Chain 5 (*DNAH5*) encodes dynein axonemal heavy chain, which is involved in ciliary assembly and cell motility and was initially found to be frequently mutated in patients with primary ciliary dyskinesia [16]. Increased incidence of non-synonymous single-nucleotide mutations and insertions/deletions was found in *DNAH2, DNAH5,* and *DNAH10* genes of CpG-island methylator phenotype positive clear cell renal cell carcinomas [17]. It has been also suggested that the *DNAH5* gene can play a role in the development of colorectal cancer [18]. Zhu et al., using the genomic and clinical data from the TCGA project, examined the impact of the somatic mutation in DNAH genes on chemotherapy response in gastric adenocarcinoma patients treated with fluoropyrimidine and platinum [19]. The mutation rates of 13 members of the DNAH gene family were higher in chemo-sensitive than in chemo-resistant patients (mutation rate in *DNAH5* gene specifically was 22.8% vs 12.5% respectively). In addition, the patients with DNAH mutations had significantly better overall survival, chemotherapy-free survival, fluoropyrimidine-free survival, and platinum-free survival. The results from TCGA analysis were validated by the authors through targeted sequencing of samples from the authors’ cohort. This confirmed association of DNAH mutations with an improved response to chemotherapy. Since the mutation in the DNAH gene affects microtubule structure and tumor cell movement, the authors speculated that it could act as a protective factor and marker of chemotherapy response. Mutation of *DNAH5* was validated by targeted sequencing, including in both pre-NACT and post-NACT samples from platinum-sensitive Case 4, suggesting it is a high confidence finding.

The role of the two other mutated genes in pre-NACT tumors of sensitive cases (*CYP2D6* and *NUTM1)* in chemotherapy response is less clear. The Cytochrome P450 Family 2 Subfamily D Member 6 (*CYP2D6*) gene encodes a member of the cytochrome P450 superfamily. The CYP2D6 enzyme is involved in the metabolism of 20-25% of clinically used drugs and is the main enzyme responsible for the conversion of tamoxifen into endoxifen, its most important metabolite [20]. Genetic variations in the *CYP2D6* gene can cause reduced enzyme activity and influence tamoxifen metabolism. However, the association between *CYP2D6* genotype and clinical outcome in patients with breast cancer treated with tamoxifen is controversial [20]. It is unclear how the mutation in *CYP2D6* could impact clinical outcome after NACT in patients with ovarian cancer, as carboplatin is excreted primarily by kidneys and paclitaxel is metabolized mainly by cytochromes P450 2C8 and 3A4 [21]. Similarly, the role of *NUTM1* in patients with ovarian cancer is unclear. The NUT Midline Carcinoma Family Member 1 (*NUTM1*) is typically expressed in normal testis and is crucial for male fertility [22]. Rearrangement in *NUTM1* has been associated with poorly differentiated carcinomas with variable squamous differentiation originating in midline organs as well as in various other neoplasms including sarcoma-like tumors, poromas, and acute lymphoblastic leukemias [22].

As mentioned, three genes were mutated exclusively in pre-NACT tumor samples of resistant cases (*CSPG4, TUBA3D, SLC35G5*). The *CSPG4* (Chondroitin sulfate proteoglycan 4) gene encodes a transmembrane protein and has been implicated in cell differentiation and migration, angiogenesis and vascularization, glial and oligodendrocyte formation and neuronal network regulation [23]. Overexpression of *CSPG4* has been found in several cancer types including triple-negative breast cancer [24], sarcomas [25], and squamous cell carcinoma of the head and neck [24]. Expression of *CSPG4* predicted poor survival and resistance to ionizing radiation in glioblastoma [26]. Somatic mutation in the *CSPG4* gene was significantly associated with PD-L1 positivity (predictor of the response to immunotherapy with PD-1/PD-L1 inhibitors) in renal cell carcinoma tumor cells in a study by Wang et al. [27]. However, the relationship of somatic mutation in the *CSPG4* gene to chemoresistance is yet to be determined. It is possible that the cells with this mutation may have been chemosensitive, since the mutation was not reflected in the surviving tumour cells.

The remaining mutated genes present in pre-NACT tumor samples from resistant cases, *TUBA3D* and *SLC35G5,* were the only two genes retained in the post-NACT tumor samples of resistant cases. The Tubulin α 3D (*TUBA3D*) gene encodes a member of the α tubulin family that heterodimerizes with β tubulin to form the main structural component of the microtubules. These are responsible for cellular structure, cell motility, transport and mitosis [28]. The expression of high levels of β3-tubulin was found to be associated with taxane resistance in ovarian cancer [29]. In contrast high expression of β3-tubulin was found to be associated with sensitivity, rather than resistance, to taxane-based chemotherapy in clear cell ovarian carcinoma by Aoki et al. [30]. However, there is no association of mutation in the *TUBA3D* gene with chemoresistance yet reported in the literature. The Solute carrier family 35 member G5 (*SLC35G5*) gene belongs to a solute carrier group of transporters, which transport organic or inorganic molecules across cell or organelle membranes [31]. However, its role in carcinogenesis has not been previously described.

We have identified candidate genes involved in the rapid development of platinum resistance in HGSC patients treated with NACT. Mutations in four genes emerged exclusively in the platinum-resistant cases following NACT (*ADGRV1, MUC17, MUC20, PAK2*). The Adhesion G Protein-Coupled Receptor V1 (*ADGRV1*) gene encodes an adhesion G-protein coupled receptor V1; its role in tumorigenesis is unclear. Auguste et al., [32] reported alterations in the *ADGRV1* gene in two of three post-chemotherapy samples but not in the chemotherapy-naïve patients with small cell carcinoma of the ovary, hypercalcemic type. Mucin 17 (*MUC17*) and Mucin 20 (*MUC 20*) genes encode high-molecular-weight membrane glycoproteins [33, 34]. Overexpression of *MUC20* was found to be a predictor of poor outcome and recurrence in colorectal cancer [35]. Low expression of *MUC20* was significantly correlated with tumour regression grade in patients with esophageal squamous cell carcinoma treated with neoadjuvant cisplatin and paclitaxel chemotherapy [36]. In contrast, the blockage of *MUC20* in in-vitro experiment contributed to increase sensitivity to paclitaxel [36]. Using HGSC genomics data from TCGA, Nabavi et al. identified somatic *MUC17* mutation at a similar frequency of platinum-sensitive and platinum-resistant groups [37]. The P21-activated protein kinase 2 (*PAK 2*) is a serine/threonine kinase, which regulates cell motility/migration, and gene transcription [38]. It has been found to promote tumor cell proliferation and survival through the AKT1 and Raf–MAPK pathways [39]. Overexpression of *PAK1* and *PAK2* has been found in ovarian cancer cells [40]. While Gupta et al. found that elevated expression of *PAK2* leads to cell proliferation and acquired chemoresistance in head and neck cancer through activation of c-Myc and PKM2 [41]. In comparison, Shuang et al., found that repression of miR-134 and leading to subsequent up-regulation of PAK2 expression contribute to paclitaxel resistance in HGSC [42]. Authors concluded that both, the miR-134 and its target *PAK2* might be a potential target for therapeutic intervention of ovarian cancer paclitaxel resistance.

The additional TCGA-OV screening [43] for the commonly mutated genes found in our study, revealed only a small number of individuals with the mutations in these genes (Table 4). While the larger numbers could have been more supportive of the results of our study, it is worth noticing that the overall sample size was also limited (*n* = 436) and much bigger sample size would be needed for positive results.

**Table 4:** The number of patients with ovarian cancer positive for the mutations in the most commonly mutated genes in our study as per the TCGA-OV data set [43]

Genes commonly mutated in our study	TCGA-OV number of individuals with mutations (out of a total of 436)
Pre-NACT platinum resistant cases	
*CSPG4*	6
*SLC35G5*	0
*TUBA3D*	1
Pre-NACT platinum sensitive genes	
*CYP2D6*	1
*NUTM1*	11
*DNAH5*	42
Post-NACT genes exclusively present in the platinum resistant cases	
*ADGRV1*	26
*MUC17*	40
*MUC20*	3
*PAK2*	8

The main limitation of our study is the small number of patients included. This is largely due to the challenge in obtaining sufficient pre and post-NACT sample from retrospectively collected cases which is largely limited by the lack of pre-NACT biopsy material. In addition, we acknowledge the lack of CRISPR-cas9 whole-genome screening in our study. While it is a powerful gene-editing tool that has been previously utilized to identify genes involved in platinum-resistance in HGSC (including but not limited to *TP53*, *p53*, *ABCB1*, *AKT1*, *ERCC1*, *EGFR*, *BRCA1*, *PIK3CA*, and *MAPK1)* [44, 45], we did not have access to this technology at the time of study conception. We believe that due to the small sample size, it could have been of a limited additional value. Despite the small number of cases, we were able to detect recurrently mutated genes in cases resistant to NACT (detected prior to treatment and/or following exposure to NACT). Although the impact of the identified mutations on function of the encoded protein is not known. The mutation could reflect a loss or gain of function or have no impact at all. In addition, mutations in non-exon regions could also have impact, particularly if these lie in regulatory (enhancer) regions. Study of a larger number of cases, preferably in a prospective setting, may reveal additional genes related to NACT response. Future studies should focus on further exploration of site-specific post-NACT changes in comparison to malignant ascites and/or plasma samples in platinum-resistant recurrence. Direct experimentation of the role of candidate genes and mutations in platinum resistance using patient-derived ascites may yield additional valuable information.

## Conclusion

In summary, our study identified genes mutated in patients with HGSC pre-NACT that are associated with platinum resistance. Additional studies are necessary to further validate these findings and identify additional genes associated with response to chemotherapy.

## Methods

### Study cases and samples

HGSC patients with a documented response to platinum-based NACT who had donated a baseline blood sample to the Princess Margaret Gynecologic Oncology BioBank and had banked tumor before and after NACT were identified. Ethics approval was obtained from the University Health Network Research Ethics Board prior to case review (protocol #14-7539). Available tumor tissue blocks from all cases were subjected to secondary pathological review. Pre-NACT (at diagnosis, prior to NACT, “Pre”) and post-NACT (at surgery after 3-5 cycles of NACT, “Post”) tumor sites were matched when possible. A total of 12 tumor blocks were selected for whole exome and/or targeted sequencing, including: a pre-NACT omental biopsy and post-NACT samples from the omentum and pelvic nodule for Case 1; a pre-NACT omental biopsy and post-NACT omental tumor for Case 2; a pre-NACT omental biopsy and post-NACT samples from the right ovary and omentum for Case 3; a pre-NACT omental biopsy and a post-NACT sample from a stomach nodule for Case 4; a pre-NACT biopsy (location not specified) and a post-NACT sample from a bowel nodule for Case 5. Tumor content was enriched to > 80% cellularity through H&E-guided macrodissection of unstained sections or tissue coring prior to DNA extraction using the Qiagen QIAamp DNA FFPE extraction kit (Qiagen, Toronto, Ontario, Canada). DNA was eluted in Buffer ATE, quantified using the Qubit Fluorometer (Life Technologies, California, USA) and quality assessed (BioAnalyzer, TapeStation, qPCR). Matched buffy coat samples were included for each case as a germline control. Platinum-resistant recurrence was defined as disease recurrence within 6 months of first-line platinum-based chemotherapy, while platinum-sensitive recurrence was defined as disease recurrence 6 months and more after completion of first-line platinum-based chemotherapy [7].

### Whole exome sequencing

Genomic DNA (200 ng) from the buffy coat, pre-NACT tumor, and post-NACT tumor samples was fragmented using a Covaris Focused-ultrasonicator, libraries were generated using the Agilent SureSelect XT Human All Exon v5 + UTR kit, and sequencing was performed on the Illumina HiSeq2000 using a 100-cycle paired-end protocol to achieve 50X (normal samples) or 100X (tumor samples) coverage. Genome Analysis Toolkit2 (GATK) Best Practices recommendations for sequencing analysis were followed. Briefly, fastqs were aligned to the reference human genome (hg19) using BWA-MEM (v0.7.12). BAM files were processed using several tools (Picard MarkDuplicates, GATK RealignerTargetCreator followed by IndelRealigner, GATK’s Base Quality Score Recalibration) and quality metrics were generated using Qualimap (v2.1). Candidate somatic mutations were identified using MuTect (v1.1.5), followed by filtering of VCF files (using VCFtools) to retain only high confidence somatic variants and annotation using ANNOVAR (v20160201).

### Deep targeted sequencing using a custom panel

A custom SureSelect XT panel was designed using the SureDesign Tool by Agilent Technologies (Mississauga, Ontario, Canada), with 1776 included regions, a total target region size of 325.797 kbp, and a total probe size of 458.215 kbp. All exons +/− 10 bp for each gene were included, with > 99% coverage of the target region. Seventy-five genes were included, based on at least one of the following criteria: 1) presence of a mutation in only platinum-resistant (or sensitive) cases; 2) detection of a mutation in > 1 resistant case; 3) detection of a mutation only in, or at an increased percentage of variant reads in, post-NACT tumor samples from platinum-resistant cases; 4) previous implication in platinum response in ovarian or other malignancies; 5) association with PFS within The Cancer Genome Atlas (TCGA) ovarian serous carcinoma dataset.

Targeted capture and sequencing on Illumina HiSeq2000 was performed using 200 ng DNA from 9 tumor and 4 matched normal samples from Cases 1-4 (See Table 1). Samples from Case 5 were not included due to insufficient remaining DNA in the pre-NACT tumor biopsy. 5000X coverage for tumor, and 100X for normal, was achieved using the 100-cycle paired-end protocol and multiplexing. Alignment of fastqs and processing of BAM files was conducted as described for whole-exome sequencing above. MuTect (v1.1.5) was used to identify candidate somatic mutations; for targeted sequencing samples, a bed file with intervals was provided. The VCF output from this step was then filtered (using VCFtools) to retain only high confidence somatic variants. Variants were annotated using ANNOVAR (v20160201). SNVs and indels were detected using Samtools mpileup (v1.2) and VarScan (v2.3.8) and copy number variants (CNVs) were identified using VarScan2 and further verified with Sequenza (v2.1.2).

## Supplementary Information

**Additional file 1.**

**Additional file 2.**

**Additional file 3.**

**Additional file 4.**

## Data Availability

The dataset used and/or analyzed during the current study are available from the corresponding authors on request.
