# Supplementary material for: Impact of neoadjuvant chemotherapy on somatic mutation status in high-grade serous ovarian carcinoma
Source: J Ovarian Res. 2022 May 2;15:50. doi: 10.1186/s13048-022-00983-5 (PMC9059396; doi:10.1186/s13048-022-00983-5)
Supplement: Supplementary file 1 — Additional file 1. [file 13048_2022_983_MOESM1_ESM.docx]

**Additional file 1. Summary of non-exonic somatic mutations by whole-exome sequencing.**

| Case # | Sample | Down-stream | Intergenic | Intronic | ncRNA | Splicing | Upstream | UTR3 | UTR5 | Shared (position)^#^ |
| --- | --- | --- | --- | --- | --- | --- | --- | --- | --- | --- |
| 1  (R) | **1-1** (pre) | 2 | 131 | 59 | 39 | 0 | 8 | 18 | 5 | **30%** (78/262) |
|  | **1-2** (post) | 87^&^ | 451 | 1752 | 254 | 1 | 69 | 1113 | 183 | **2%** (78/3917) |
| 2  (R) | **2-1** (pre) | 12 | 115 | 92 | 32 | 1 | 9 | 38 | 10 | **36%** (111/311) |
|  | **2-2** (post) | 14 | 147 | 124 | 43 | 0 | 6 | 80 | 8 | **26%** (111/423) |
| 3  (R) | **3-1** (pre) | 7 | 113 | 111 | 48 | 1 | 11 | 34 | 8 | **45%*** (150/334) |
|  | **3-2** (post) | 3 | 120 | 90 | 43 | 0 | 4 | 23 | 6 | **43%**** (124/290) |
|  | **3-3** (post) | 1 | 103 | 68 | 35 | 1 | 3 | 28 | 2 | **31%**** (75/243) |
| 4  (S) | **4-1** (pre) | 4 | 143 | 151 | 34 | 1 | 4 | 64 | 7 | **31%** (129/411) |
|  | **4-2** (post) | 5 | 117 | 128 | 40 | 3 | 9 | 59 | 10 | **35%** (129/373) |
| 5  (S) | **5-1** (pre) | 25 | 138 | 406 | 69 | 5 | 21 | 278 | 33 | **12%** (118/976) |
|  | **5-2** (post) | 16 | 242 | 169 | 59 | 2 | 8 | 88 | 10 | **20%** (118/597) |

***LEGEND***

Counts for mutation types other than exonic are shown, in addition to the overlap of mutations in pre-NACT and post-NACT samples from the same patient. ^#^comparison made based on genomic position. *mutations shared with at least one post-NACT sample from same case. **mutations shared with pre-NACT sample from same case. ^&^additional genes classified as “upstream/downstream” not shown in table but included in comparison of pre- and post-NACT samples. “R” = platinum-resistant; “S” = platinum-sensitive
