# Supplementary material for: Impact of neoadjuvant chemotherapy on somatic mutation status in high-grade serous ovarian carcinoma
Source: J Ovarian Res. 2022 May 2;15:50. doi: 10.1186/s13048-022-00983-5 (PMC9059396; doi:10.1186/s13048-022-00983-5)
Supplement: Supplementary file 2 — Additional file 2. [file 13048_2022_983_MOESM2_ESM.docx]

**Additional file 2. Amino acid changes for all recurrently mutated genes by whole-exome sequencing.**

| Gene Symbol | Case 1 (R) | | Case 2 (R) | | Case 3 (R) | | Case 4 (S) | | Case 5 (S) | |
| --- | --- | --- | --- | --- | --- | --- | --- | --- | --- | --- |
|  | **Pre** | **Post** | **Pre** | **Post** | **Pre** | **Post** | **Pre** | **Post** | **Pre** | **Post** |
| *Genes mutated in >1 platinum-resistant cases only (N=16)* | | | | | | | | | | |
| *ADGRV1/  GPR98^3^ |  | L2004F R2097C |  | R2090H |  |  |  |  |  |  |
| *AOC1/  ABP1^3^ |  | T16M |  |  | R197H | R197H^6^ |  |  |  |  |
| ARHGAP5 |  |  | C972Y/  G2915A |  |  | E489K^6^ |  |  |  |  |
| *CSPG4^1^ | R450W |  | E441K | E441K |  |  |  |  |  |  |
| KIR2DL1 |  | T91K V111L H203R R266C T333A |  |  | P21T |  |  |  |  |  |
| KRTAP4-11 | R66H |  |  |  |  | V46M^6^ |  |  |  |  |
| MMP9 |  | P133L | P133L |  |  |  |  |  |  |  |
| *MTMR11^3^ |  | M87V/ M159V |  |  | K135T/ K207T | K135T/ K207T^5^ |  |  |  |  |
| MUC17^3^ |  | K227Q P571L T959A P978L S1083P S1097R S1242T L1348P V1480A S3299N |  |  |  | V2771L^6^ |  |  |  |  |
| MUC20^3^ |  | T271I/  T290I/  T442I G343R/ G362R/ G514R |  |  |  | G201S/  G220S/ G372S^6^ |  |  |  |  |
| *OR52N5^3^ |  | V133I | A128D | A128D |  |  |  |  |  |  |
| PAK2^3^ |  |  |  | K128R |  | Q101H^6^ |  |  |  |  |
| *PCDHB11^3^ |  |  | D410Y | D410Y |  | G532S^6^ |  |  |  |  |
| *TMEM14B^3^ |  | R74C/  R97C/ R108C | R74C/ R97C/ R108C | R74C/ R97C/ R108C |  |  |  |  |  |  |
| TTN |  | T765I/ T811I G9378R/  G10305R/ G10622R  R9852H/ R10779H/ R11096H | S4477R/ S5404R/ S5721R |  |  |  |  |  |  |  |
| USP8 |  | T679A/ T785A | N658K/ N764K |  |  |  |  |  |  |  |
| *Genes mutated in >1 platinum-sensitive cases only (N=5)* | | | | | | | | | | |
| *CYP2D6^2^ |  |  |  |  |  |  | R278L/ R329L |  | P34S R314H/ R365H |  |
| *DNAH5^2^ |  |  |  |  |  |  | R2677X | R2677X | S1798Y |  |
| FAM186A |  |  |  |  |  |  |  | A1310V | M2193I |  |
| MACF1 |  |  |  |  |  |  |  | G2693A | S4670T |  |
| NUTM1^2^ |  |  |  |  |  |  | L471F/ L489F/ L499F |  | Q21R |  |
| *Genes mutated in 5/5 cases (N=1)*** | | | | | | | | | | |
| MUC2 | T1580N |  | P1629T T1705I | P1629T T1705I |  | T1704I^6^ | P1675T |  | T1597I T1705I | G648C T1580N T1705I |
| *Genes mutated in 4/5 cases (N=3)*** | | | | | | | | | | |
| DDX11 |  | A798V/ A822V/ A848V | P922R |  |  |  | A581P/ A607P | A581P/ A607P |  | R160W/ R186W |
| *TP53 | S88F/ S127F | S88F/  S127F | Q13X/ Q52X | Q13X/ Q52X | R147X/ R174X/ R267X/ R306X | R147X/ R174X/  R267X/ R306X^6^ | R54X/ R81X/ R174X/ R213X | R54X/  R81X/ R174X/ R213X |  |  |
| *TUBA3D^1,4^ |  | Y432C | G162S R221S | G162S | A126V | R221S^6^ |  | Y432C |  |  |
| *Genes mutated in 3/5 cases (N=9)*** | | | | | | | | | | |
| CACNA1S^3^ |  | L458H |  | G628S |  |  | R794C |  |  |  |
| CDC27 |  | C562S/  C622S/ C623S/ C629S |  |  |  |  | C562S/ C622S/ C623S/ C629S/ | H548N/  H608N/ H609N/  H615N |  | I174T/ I235T A212G/ A273G |
| FLG |  | T454A R1891Q  S2045T  Y2194H H2507Q  G2545R D2936G | H980D | H980D |  |  |  |  |  | E953Q |
| FRG1 | I157V | S26N |  |  |  | S26N^6^ | S26N | S26N |  |  |
| IGFN1 |  | G1655S |  |  |  |  |  | T1549R | M2173I | M2173I |
| *KIR2DL3 |  | V9A  P21T T221I |  |  |  | R290S^6^ |  |  | N307D | N307D |
| SLC35G5^1,4^ | W103R | W103R | S114N | S114N |  |  |  |  |  | R90H W103R |
| ZNF208 |  |  | K668E |  | K668E |  | K668E |  |  |  |
| ZSCAN31 |  | K46R/ K205R | E144D |  |  |  |  |  | T50S |  |
| *Additional genes mutated in 2/5 cases (N=50)*** | | | | | | | | | | |
| ACCS |  | D59N  P421L |  |  |  |  |  | E240D |  |  |
| C10orf113 |  | D110H |  |  |  |  |  |  | D110H |  |
| C19orf40 |  | I97T/  I192T |  |  |  |  | K74N |  |  |  |
| C6orf10 |  | S211P/  S225P/ S227P |  |  |  |  |  |  | I299V/  I313V/ I315V |  |
| C7orf72 |  | T268A |  |  |  |  |  |  | H91Y | H91Y |
| CFAP46 |  | G2184S |  |  |  |  |  |  | A815S |  |
| CHUK |  | P685L |  |  |  |  |  |  | V268I |  |
| CUBN |  |  | V2122I |  |  |  | V2017M |  |  |  |
| CX3CR1 |  | F8L |  |  |  |  | F12I/  F44I |  |  |  |
| DPCR1 |  | E720G |  |  |  |  |  | G502E |  |  |
| EBLN1 |  | G149R |  |  |  |  |  |  | M235T |  |
| FAM185A |  | R230C/  R347C |  |  |  |  |  |  | R230C/  R347C |  |
| FBN3 |  | L1904F |  |  |  |  |  |  | E2610D |  |
| GOLGA6L2 |  |  |  |  |  | V797A^6^ |  |  |  | V797A |
| HELZ2 |  | S338L S219N/  S788N  R812K/  R1381K  R1083Q/  R1652Q  Q1480E/  Q2049E  T1601M/ T2170M |  |  |  |  |  |  | L994V/  L1563V | L994V/  L1563V |
| IFITM3 | P70T |  |  |  |  |  | P70T | P70T |  |  |
| KHDC3L |  | A201G |  |  |  |  |  |  | A201G |  |
| KIF20B |  | K1609E/ K1649E |  |  |  |  |  |  | H749L/ H789L |  |
| KIR3DL1 |  | S79G  V113M  G159W  P184S  L187R |  |  |  |  |  |  | S79G |  |
| KLHDC9 |  | S288N/  A282T |  |  |  |  |  |  | S171R |  |
| KRTAP9-8 |  |  |  |  | I60T | I60T^6^ |  | I60T |  |  |
| LRRK2 |  |  |  |  | Q103K |  | I1513M |  |  |  |
| MUC16 |  | Q9405R |  |  |  |  |  |  |  | T12180S |
| MUC4 |  |  |  | P1680S P1962S  S3370T  S3448L  S4199Y |  |  |  |  | N215K/  N266K/  N4502K | P2192S L3094F  A3737S  H3741Q |
| MUC5B |  | T4686M |  |  |  |  |  |  | V3909I | V3909I |
| NBPF8 |  |  | E676K | E676K |  |  |  |  |  | G628E |
| NEB |  | I6534V/  I8402V/  I8437V  W3348C/  W3603C |  |  |  |  |  |  | K1027N |  |
| OBSCN |  | Q502R  A1532V/  A1716V  V2720M/  V3149M  A3300T/  A3729T |  |  |  |  |  |  | R6984Q/  R7941Q |  |
| OR7C1 |  | S99G S210P |  |  |  |  |  |  | S210P |  |
| PARP4 |  |  | T935P | T935P |  |  |  |  | V458I | V458I |
| PCDHA6 |  |  |  |  | G97R | G97R^5^ |  |  | G97R | G97R |
| PEAK1 |  | S1542T |  |  |  |  | E436X | E436X |  |  |
| PKD1L2 |  | G100C/  G785C |  |  |  |  |  |  | E221G |  |
| PNMAL2 |  |  | G184A |  |  |  |  |  | A599S |  |
| RGSL1 |  | W256C |  |  |  |  | A298S | A298S |  |  |
| RP1L1 |  | G514S |  |  |  |  |  |  | E2171K  L792P |  |
| SAG |  | V403A |  |  |  |  |  |  | V403I |  |
| SETD8 |  |  |  |  |  | Q155P^6^ | R238P | R238P |  |  |
| SLC22A20 |  | E82K |  |  |  |  |  |  | E82K |  |
| SLC28A1 |  | D521N |  |  |  |  |  |  | A190S |  |
| SNAPC4 |  | H799Q |  |  |  |  |  |  | P1295T | P1295T |
| SP6 | A195D |  |  |  |  |  | S298L | S298L |  |  |
| STEAP1B |  | I210T/  I229T |  |  |  |  |  |  | K185E/  K204E |  |
| STEAP2 |  | Y284C |  |  |  |  |  |  | R456Q |  |
| TNS1 |  | R1004W |  |  |  |  |  |  | C94F |  |
| TUBG2 |  | M413V |  |  |  |  |  |  | M413V |  |
| UGT1A6 |  | S7A |  |  |  |  |  |  |  | G173C |
| ZFYVE9 |  | K134N |  |  |  |  |  |  | K134N |  |
| ZNF28 |  |  |  |  |  | E639G^5^  R589Q |  |  | E639G | E639G |
| ZNF488 |  | A72V |  |  |  |  |  |  | A72V |  |

*included on targeted panel

**not related to NACT response status

**^1^**gene mutated in pre-NACT samples from >1 resistant cases but not sensitive cases (*CSPG4, SLC35G5, TUBA3D*)

**^2^**gene mutated in pre-NACT samples from both sensitive cases but not resistant cases (*CYP2D6, NUTM1, DNAH5*)

**^3^**gene mutated in post-NACT samples from >1 resistant cases but not sensitive cases (*ADGRV1*, *AOC1*, *CACNA1S*, *MTMR11*, *MUC17*, *MUC20*, *OR52N5*, *PAK2*, *PCDHB11*, *TMEM14B*)

**^4^**gene mutated in both pre and post-NACT samples from >1 resistant cases but not sensitive cases (*SLC35G5, TUBA3D*)

**^5^**gene mutated in both omental and ovarian post-NACT samples from Case 3 *(MTMR11, PCDHA6, ZNF28)*

^6^gene mutated in only 1 post-NACT sample from Case 3
