# Supplementary material for: Impact of neoadjuvant chemotherapy on somatic mutation status in high-grade serous ovarian carcinoma
Source: J Ovarian Res. 2022 May 2;15:50. doi: 10.1186/s13048-022-00983-5 (PMC9059396; doi:10.1186/s13048-022-00983-5)
Supplement: Supplementary file 3 — Additional file 3. [file 13048_2022_983_MOESM3_ESM.docx]

**Additional file 3. Comparison of non-synonymous exonic mutations in Case 3 by treatment status and site by whole-exome sequencing.**

| Gene Symbol | Pre-NACT  (omental biopsy) | Post-NACT  (omentum) | Post-NACT  (ovary) |
| --- | --- | --- | --- |
| *Genes mutated in all samples (N=8)* | | | |
| *CDK12 | R882W (31/114, 27%)** | R882W (11/243, 5%) | R882W (79/179, 44%) |
| *FOXJ1 | V120E (44/90, 49%) | V120E (14/149, 9%) | V120E (52/100, 52%) |
| *HMCN1 | G1738R (39/191, 20%) | G1738R (12/228, 5%) | G1738R (53/235, 23%) |
| *MMRN1 | Q468X (116/292, 40%) | Q468X (21/225, 9%) | Q468X (171/401, 43%) |
| *MTMR11 | K135T/K207T  (23/95, 24%) | K135T/K207T  (7/121, 6%) | K135T/K207T  (21/113, 19%) |
| PCDHA6 | G97R (7/72, 10%) | G97R (12/104, 12%) | G97R (5/65, 8%) |
| *RBM12 | L667F(13/62, 21%) | L667F (5/90, 6%) | L667F (19/71, 27%) |
| *REV3L | S1387I/S1465I  (79/289, 27%) | S1387I/S1465I  (14/311, 5%) | S1387I/S1465I  (86/385, 22%) |
| *Genes mutated in both post-NACT samples but not pre-NACT sample (N=2)* | | | |
| OR10G9 |  | I28V (9/122, 7%) | I28V (7/157, 4%) |
| ZNF28 |  | R589Q (16/277, 6%)  E639G (43/371, 12%) | E639G (15/254, 6%) |
| *Genes mutated in pre-NACT and omental post-NACT sample (N=6)* | | | |
| C11orf24 | T134M (12/31, 39%) | T134M (4/32, 13%) |  |
| KRTAP9-8 | I60T (3/33, 9%) | I60T (4/51, 8%) |  |
| OSMR | K269E (23/100, 23%) | K269E (6/136, 4%) |  |
| SPP2 | R201W (73/273, 27%) | R201W (14/284, 5%) |  |
| TPTE2 | N118D/N336D/N370D/N447D (8/120, 7%) | N118D/N336D/N370D/N447D (11/208, 5%)  Y120F/Y338F/Y372F/Y449F (14/223, 6%) |  |
| *TUBA3D | A126V (3/12, 25%) | R221S (4/8, 50%) |  |
| *Genes mutated in pre-NACT and ovarian post-NACT sample (N=13)* | | | |
| * AOC1 | R197H (32/101, 32%) |  | R197H (41/119, 34%) |
| *BEND2 | P565R/P656R  (63/163, 61%) |  | P565R/P656R  (59/170, 35%) |
| *CDAN1 | R1015H (16/50, 32%) |  | R1015H (24/76, 32%) |
| *CRYZL1 | E121K (23/148, 16%) |  | E121K (52/205, 25%) |
| *GRM5 | V313E (44/189, 23%) |  | V313E (50/223, 22%) |
| *HSPB7 | P93H (25/81, 31%) |  | P93H (29/102, 28%) |
| LOC729159 | T148M (8/22, 36%) |  | T148M (7/15, 47%) |
| *LRP1 | A1727V (21/105, 20%) |  | A1727V (28/105, 27%) |
| OR6C3 | Y256C (71/290, 24%) |  | Y256C (56/293, 19%) |
| *PLD5 | V18M/V164M/V226M  (16/84, 19%) |  | V18M/V164M/V226M  (20/101, 20%) |
| *TEP1 | G2298V (38/134, 28%) |  | G2298V (36/128, 28%) |
| *TP53 | R147X/R174X/R267X/  R306X (43/106, 41%) |  | R147X/R174X/R267X/  R306X (39/122, 82%) |
| *TRIP11 | E1545Q (59/264, 22%) |  | E1545Q (86/329, 26%) |
| *Genes mutated in omental post-NACT sample only (N=14)* | | | |
| ARHGAP5 |  | E489K (7/173, 4%) |  |
| ARMC4 |  | D425Y (7/71, 10%) |  |
| CLCNKA |  | A244V/A287V (8/52, 15%) |  |
| FRG1 |  | S26N (7/141, 5%) |  |
| KRTAP4-11 |  | V46M (6/87, 7%) |  |
| MUC17 |  | V2771L(9/254, 4%) |  |
| MUC2 |  | T1704I (11/74, 15%) |  |
| NUP50 |  | T95A/T123A (9/265, 3%) |  |
| PAK2 |  | Q101H (5/98, 5%) |  |
| *PCDHB11 |  | G532S (3/11, 3%) |  |
| PLEC |  | E1839D/E1857D/E1849D/  E1871D/E1875D/E1898D/  E2008D (6/90, 7%) |  |
| SGSM2 |  | R213H (3/23, 13%) |  |
| TBC1D3B/F |  | H156D (4/13, 31%) |  |
| UGT2B11 |  | I442V (3/40, 8%) |  |
| *Genes mutated in ovarian post-NACT sample only (N=10)* | | | |
| CFAP47 |  |  | D303E/D2857E (6/124, 5%) |
| GOLGA6L2 |  |  | V797A (3/5, 60%) |
| *GPR101 |  |  | E322K (39/215,18%) |
| *KIR2DL3 |  |  | R290S (4/18,22%) |
| MUC20 |  |  | G201S/G220S/G372S  (6/114, 5%) |
| PCDHGB6 |  |  | V46M (8/95, 8%) |
| PGAM1 |  |  | A3S (6/61, 10%) |
| SETD8 |  |  | Q155P (5/93, 5%) |
| *SPATA31D1 |  |  | P154S/P154L (29/180, 16%) |
| TRIM49 |  |  | N298S (11/126, 9%) |

*included on targeted panel; **allele fraction of mutation (variant allele/reference allele)
