# Supplementary material for: Impact of neoadjuvant chemotherapy on somatic mutation status in high-grade serous ovarian carcinoma
Source: J Ovarian Res. 2022 May 2;15:50. doi: 10.1186/s13048-022-00983-5 (PMC9059396; doi:10.1186/s13048-022-00983-5)
Supplement: Supplementary file 4 — Additional file 4. [file 13048_2022_983_MOESM4_ESM.docx]

**Additional File 4. Genes selected for targeted deep sequencing.**

***LEGEND***

The presence of a somatic non-synonymous exonic mutation in a gene in a given sample by whole-exome sequencing (50-200X) is indicated using a colored box, with the relative percentage of variant reads in pre-NACT and post-NACT tumor samples indicated by color: purple = mutation detected only in pre-NACT tumor; green = ≥10% higher in pre-NACT versus post-NACT tumor; black = similar variant percentage in pre-NACT and post-NACT tumor; red = ≥10% higher in post-NACT versus pre-NACT tumor; blue = mutations detected only in post-NACT tumor. The number of samples from the TCGA HGSOC dataset with a mutation in a given gene is indicated at right.
